# A novel simplified approach for endodontic retrograde surgery in short single-rooted teeth

**DOI:** 10.1186/s12903-024-03879-6

**Published:** 2024-01-31

**Authors:** Chen Zheng, Wenzhi Wu, Yulian Zhang, Zhenhang Tang, Zhijian Xie, Zhuo Chen

**Affiliations:** Stomatology Hospital, School of Stomatology, Zhejiang University School of Medicine, Zhejiang Provincial Clinical Research Center for Oral Diseases, Key Laboratory of Oral Biomedical Research of Zhejiang Province, Cancer Center of Zhejiang University, Engineering Research Center of Oral Biomaterials and Devices of Zhejiang Province, Hangzhou, 310000 China

**Keywords:** Endodontic microsurgery, Apical sealing, µCT, Orthograde obturation, Retrograde obturation

## Abstract

**Background:**

High technical thresholds, long operative times, and the need for expensive and specialized equipment impede the widespread adoption of endodontic microsurgery in many developing countries. This study aimed to compare the effects of a simplified, cost-effective, and time-efficient surgical approach involving orthograde obturation using biological ceramic material greater than 6 mm combined with apicoectomy for single-rooted teeth with short lengths with those of the conventional and current standard methods.

**Materials and methods:**

Forty-five premolars equally categorized into three groups: conventional surgery group, standard surgery group, and modified surgery group. A µCT scan was used to calculate the volume of voids. A micro-leakage test and scanning electron microscope (SEM) were performed to assess the sealing effect. Additionally, four cases of chronic periapical periodontitis in the anterior region were selected, and the patients received either the modified approach or the standard surgery for endodontic microsurgery.

**Results:**

The volumes of voids in the apical 0–3 mm of the modified group and the standard group were comparable. The micro-leakage test and SEM examination demonstrated closely bonded fillings in the dentinal walls in both the modified surgery group and standard surgery group. The outcomes of the preliminary application of this modified procedure on patients were successful at the time of the follow-up cutoff.

**Conclusions:**

The modified surgery group exhibited similar root canal filling and apical sealing abilities with the standard procedure for single-rooted teeth with short lengths (< 20 mm). The preliminary application of this modified surgical procedure achieved favorable results.

**Supplementary Information:**

The online version contains supplementary material available at 10.1186/s12903-024-03879-6.

## Introduction

Apical surgery/microsurgery is now considered a predictable treatment option for refractory apical periodontitis, while non-surgical approaches are contraindicated or inefficacious [1]. Conventional apical surgery includes root-tip resection, apical curettage, enlargement, and sealing of apical foramina [2]. Recently, because of advancements in operating microscopes, bioceramic materials, ultrasonic devices, and microsurgical instruments, endodontic microsurgery has emerged as a prominent intervention [3]. A meta-analysis study has reported a significantly higher success rate of 94% for microsurgery compared to the group that underwent conventional apical surgery (with a success rate of approximately 60%) [4]. Especially, advances in root-end filling material are essential for improving the outcomes of apical surgery/microsurgery [5]. Bioceramic materials, such as mineral trioxide aggregate (MTA) and iRoot BP, exhibit better biological and physico-chemical properties along with biocompatibility than traditional sealing materials [6]. The contact interface of bioceramic materials and tissue form a mineralized layer, which creates a biological seal between the materials and the dentine interface [7]. Although microsurgical techniques have overcome many technical limitations of conventional surgery, such as poor visibility and insufficient infection clearance, several challenges still require resolution.

First, endodontic microsurgery relies on expensive and specialized equipment and is highly technically sensitive. In many developing countries, the usage rate of dental operating microscopes (DOM) is low. A questionnaire survey conducted in the Middle East revealed the use of DOM by endodontists was below 50%, and the reasons for not using DOM were positional difficulties and prolonged treatment time [8]. Furthermore, the knowledge and practical experience regarding microsurgical techniques among general dental practitioners and specialist endodontists in developed and developing nations are imbalanced [9, 10]. Second, the long operative time is one of the key factors restraining the popularity of endodontic microsurgery. Prolonged surgical time will lead to a more severe inflammatory response, an increased risk of infections, and reduced patient satisfaction [11–13]. In addition, the formation and propagation of microcracks after ultrasonic root-tip preparation, which may lead to vertical root fractures, is of great concern [14]. Previous studies suggest ultrasonic root-tip retropreparation in apical microsurgery can propagate preexisting dentinal defects [15, 16].

Therefore, to address the aforementioned problems, we proposed a new surgical technique involving orthograde obturation using biological ceramic material greater than 6 mm combined with an apicoectomy. This approach aims to optimize the operation difficulty, improve surgical prognosis, reduce operation time, and reduce patient’s fear. Because of the lack of the step of reverse root-tip preparation, this surgical procedure uses strict indications, only applicable for single rooted-teeth with short lengths (< 20 mm). Anterior teeth, which typically have only one root canal, tend to exhibit high healing rates after surgery because of their simpler anatomy [2, 17]. The 20-mm limit in tooth length is imposed considering the difficulties in orthograde filling.

This study aimed to evaluate the filling effect and interfacial microleakage using the novel simplified approach and conducted a primary evaluation of the clinical application of this procedure. We hypothesized that this simplified surgical procedure would demonstrate comparable filling and apical sealing abilities with the standard procedure.

## Materials and methods

After obtaining approval from the Ethical Committee of the Stomatology Hospital, Zhejiang University School of Medicine (Approval No. 2019-39), 45 premolars were acquired from Zhejiang Stomatology Hospital. These premolars, with no obvious root canal cracks, were extracted recently for orthodontic reasons. The morphology of root canals was assessed using buccolingual and mesiodistal radiographs. The inclusion criteria for teeth selection were as follows: single-rooted teeth with type I canal system according to Vertucci’s classification and the crown-to-root ratios after apicoectomy of 3 mm should not exceed 1:1. Multiple-canal teeth, teeth with root fractures, cracks, or perforations, root caries, resorptions, and previous root canal treatment were excluded. The length of each tooth was standardized into 20 mm by horizontally flattening the crown.

### **Sample preparation before** in vitro **simulated surgery**

The pulp chamber of each premolar was opened using a diamond bur (TF-12, Mani Inc, Tochigi, Japan). To check apical patency and the working length (WL), a hand K-type file (15# K-file, Dentsply Sirona, Ballaigues, Switzerland) was introduced into the canal until the tip was visible at the foramen. The WL was set as 1 mm short of this length. All canals were instrumented using a #35 WaveOne Gold file (Dentsply Sirona) accompanied by the irrigation with 17% ethylenediamine tetraacetic Acid (EDTA, Pulpdent Corp, Watertown, America) and 3% sodium hypochlorite (NaCLO, Langli lnc, Wuhan, China) according to the irrigation method by Lee et al. [18]. A final irrigation was applied using 10 mL 3% NaOCl (1.0 mL min^− 1^) accompanied with passive ultrasonic irrigation. Then the root canals were rinsed with 5 mL 0.9% NaCl. The root canals were dried with dental absorbent points (Gapadent, Scs, Foshan, China). These teeth were then randomly assigned into three groups: (1) conventional surgery group, where the full length of teeth was obturated with #35/0.06 hot gutta-percha and iRoot-SP root canal sealer (Innovative Bioceramix, Vancouver, BC, Canada) using warm gutta-percha obturation by B&L SuperEndo Alpha II heat plugger and SuperEndo beta Gutta percha Heating System (B&LBiotech, Gyeonggi-do, SouthKorea); (2) standard surgery group, where the root canals were filled via the same methods as the conventional group; and (3) modified surgery group, where iRoot-BP Plus (Innovative Bioceramix) was incrementally inserted and compacted apically according to the working length with vertical pressurizers, and the minimum filling thickness was 6 mm. Radiographs were taken to verify the quality of the filling. If the plug was poorly placed or had too many voids, it was removed with ultrasonic tips. Then a cotton pellet dipped in 0.9% saline was placed on top of the filling materials. After 24 h, the remaining part of each canal was obturated with hot gutta-percha. For all three groups, the access cavities were sealed with composite resin (Filtek™ Z350 XT, 3 M, Shanghai, China). Subsequently, the samples were stored at 37℃ for 7 days in 100% humidity. All the above procedures were performed by an operator under the assistance of a surgical microscope (Kinevo 900. Carl Zeiss, Gottingen, Germany) at 10× magnification. Instruments were changed for every five samples.

### In vitro **simulated surgical procedure**

**Fig. 1:**
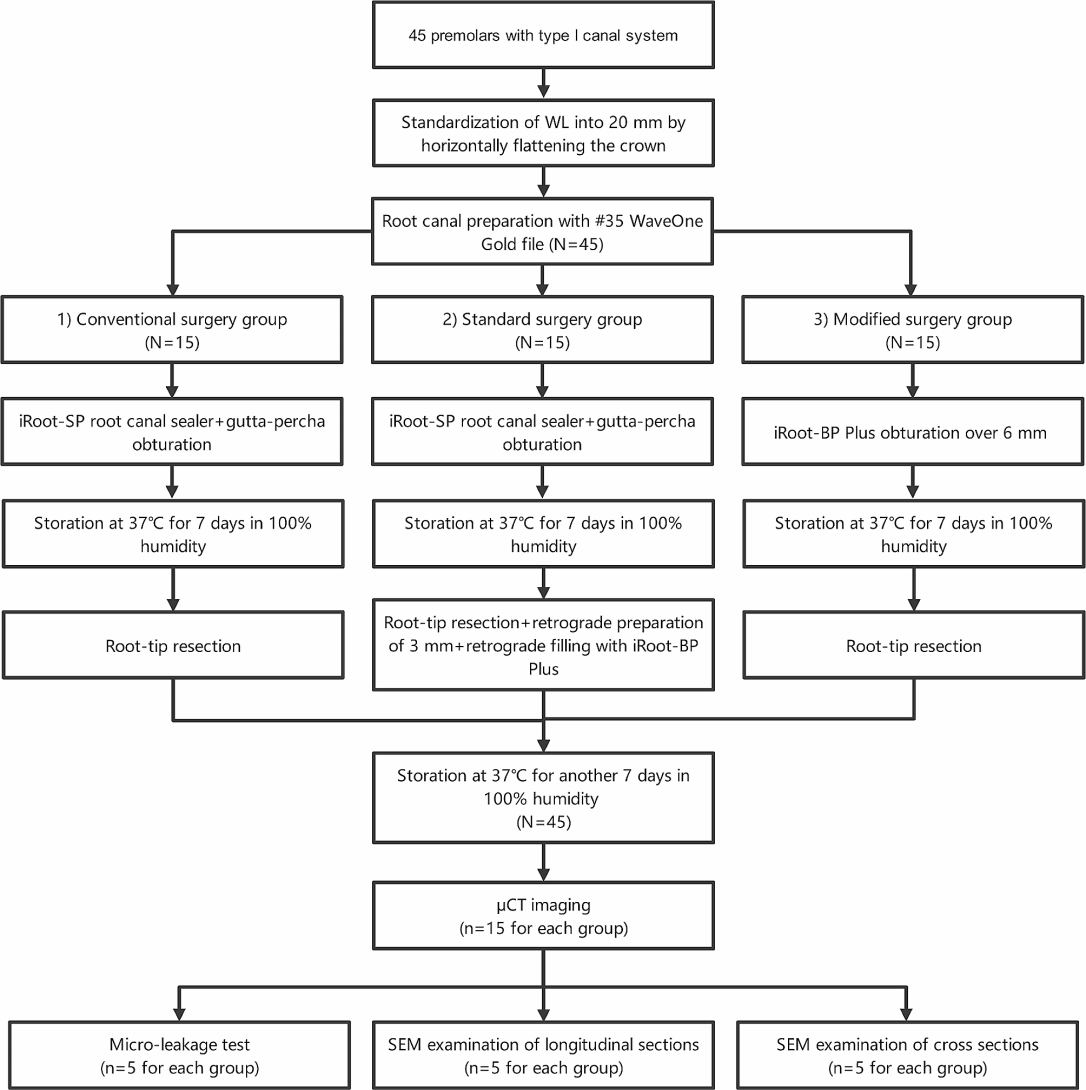
Schematic drawing of the flow of methodology used in the present study

All samples were resected 3 mm from the apex with a fissure bur at a 90-degree angle. Only the standard surgery group received a retrograde preparation of 3 mm using ultrasonic tips, and the apical cavity was retrogradely filled with iRoot BP. Subsequently, all samples were stored at 37℃ for additional 7 days in 100% humidity to ensure the filling materials were sufficiently solidified. The flow of the treatment performed is shown in a schematic drawing (Fig. 1).

### µCT imaging

All samples were scanned using µCT with Milabs U-CT-XUHR (U-SPECT, MILabs, Heidelberglaan, Netherlands) to calculate the presence and volume of voids (the space between the dentin walls and the root-end obturation material or within the obturation material). The percentage of porosity was also analyzed. Specimens were scanned at scanning parameters of(116 kV, 60 µA, 8 μm). After image acquisition, Milabs software (version 1.4.4) was used to reconstruct the digital data and distinguish dentin, iRoot-BP, and gutta-percha with different grayscale values. Before calculating the volume of voids, the region of interest (ROI) was selected for volume rendering. The region was delineated using the 3D selection tool.

### Micro-leakage test and SEM

Five samples from each group, which have recieved in vitro simulated surgical procedure, were evenly smeared with nail polish on the peripheral wall of the upper root, 1 mm from the apex, twice. The samples were placed in a dry environment to cure nail polish at 37 ℃ for 24 h. Subsequently, periapical dentin with a 6-mm section was immersed in a 2% methylene blue solution at 37℃ and 100% humidity for 72 h. Afterward, all samples were rinsed thoroughly with water, and the nail polish was scraped off with a scraper.

For SEM, five teeth from each group were longitudinally cut with a single emery wheel on a precision microtome. Another 5 samples from each group were crossly cut using the same method and fixed overnight with 1.25% glutaraldehyde solution. Dehydration was performed in a graded ethanol series. A cold field emission scanning electron microscope (SU8010, Hitachi Ltd., Tokyo, Japan) was used to observe the junction zone of the filling material and the inner dentinal wall.

### Clinical case analysis

The clinical protocol was approved by the Ethical Committee of the Stomatology Hospital, Zhejiang University School of Medicine (Approval No. 2020-03 (R)-No. 98). Four cases of chronic periapical periodontitis in the anterior region were selected and received either the modified approach or the standard surgery for endodontic microsurgery. Informed consent was obtained from all patients. The treatment procedures and the examination results were recorded. Digital periapical film and cone-beam computed tomography (Scanora® 3D system, Soredex Oy, Tuusula, Finland)) scans were taken after surgery and during follow-up.

### Statistical analysis

The Shapiro–Wilk and Levene tests were used to assess the normality of the distribution and the equality of variances. One-way analysis of variance and the Bonferroni multiple comparison tests were used. The level of significance was set at *P*<0.05.

## Results

### Volumes of filling voids

**Fig. 2:**
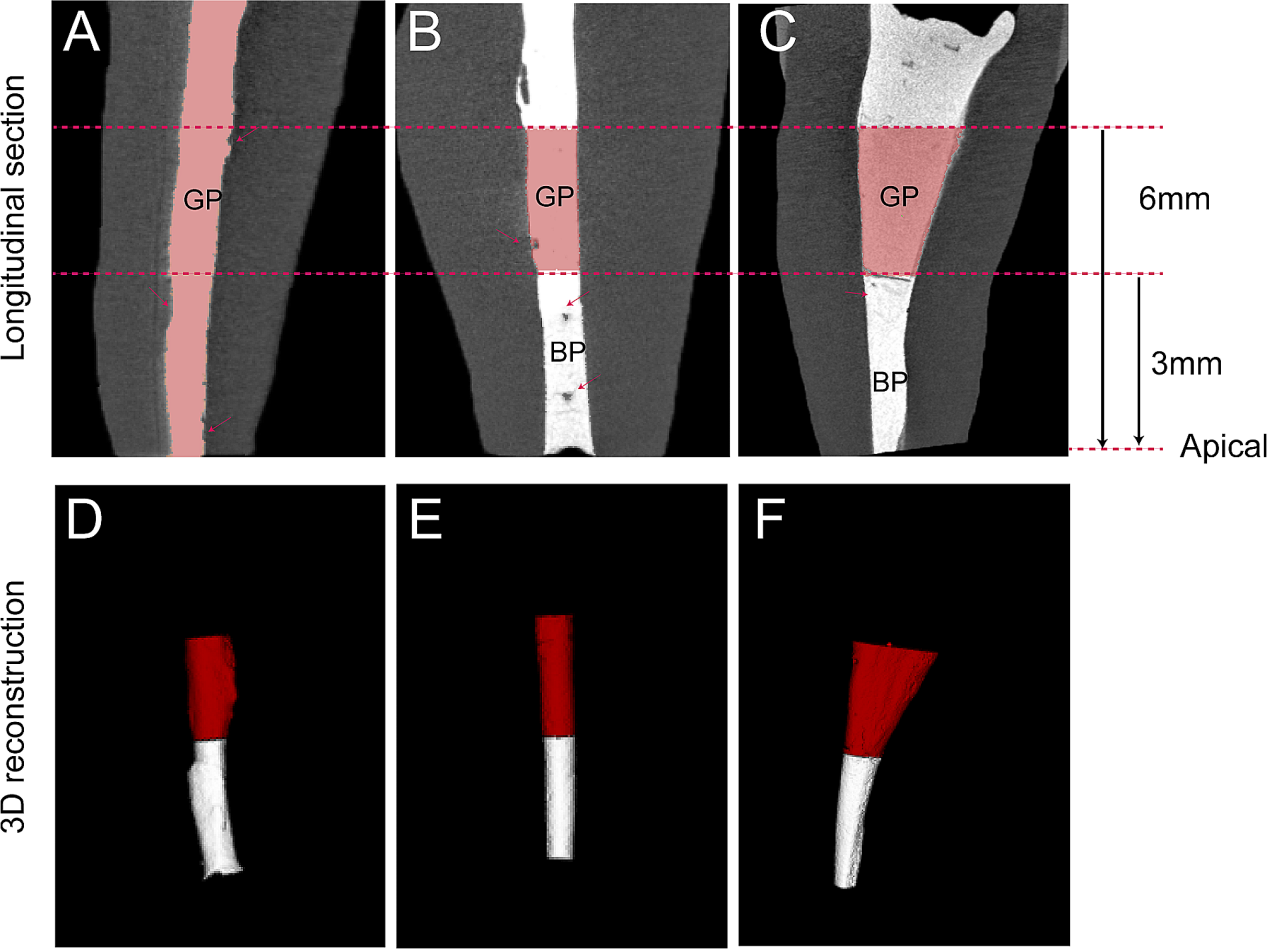
Representative images of µCT data. **(A–C)** Sagittal images of samples; red arrow indicates voids. **(D-F)** 3D reconstruction images of fillings in the root canal; the red part is the apical 3–6 mm, and the white part indicates the apical 0–3 mm filling materials in the root canal. **(A and D)** Samples from the conventional surgery group. **(B and E)** Samples from the standard surgery group. **(C and F)** Samples from the modified surgery group. GP, gutta-percha; BP, iRoot BP.

The µCT data of all three groups were reconstructed and analyzed. Sagittal images of samples were shown in Fig. 2A–C. Representative 3D reconstruction images of the apical 6 mm root canal after in vitro simulated surgical procedure were shown in Fig. 2D–F. In the conventional surgery group, voids appeared mostly between the fillings and the dentinal walls because of the application of the warm gutta-percha obturation technique (Fig. 2A). In the standard surgery group, voids appeared mostly inside the fillings, which may be attributed to insufficient compaction during retrograde filling of iRoot BP (Fig. 2B).

The total volumes of space in root canals, the total volumes of fillings, and the total void volumes in apical 0–3 mm and 3–6 mm root segments were measured, and the total void ratios were calculated. The results are presented in Table 1. No significant differences were noted in the total volume of space and fillings in apical 0–3 mm and 3–6 mm among the three groups, suggesting comparability among groups. The volume of voids in apical 0–3 mm was 0.0077 ± 0.011 mm^3^ in the modified surgery group and 0.0062 ± 0.041 mm^3^ in the standard surgery group. No difference was found between the modified surgery group and the standard surgery group; however, the volumes and ratios of voids in apical 0–3 mm were significantly greater in the conventional surgery group than in the other two groups.

**Table 1:** Distribution of filling voids: Mean values and standard deviations in the apical 6 mm of the resected root

	Modified surgery group	Standard surgery group	Conventional surgery group
Total volume of apical 3 mm (mm^3^)	1.55 ± 0.96	1.46 ± 0.74	1.43 ± 0.70
Total volume of filling of apical 3 mm (mm^3^)	1.54 ± 0.95	1.45 ± 0.74	1.41 ± 0.70
Total volume of voids of apical 3 mm (mm^3^)	0.0077 ± 0.011^a^	0.0062 ± 0.041^a^	0.019 ± 0.011^b^
Total volume of apical 3-6 mm (mm^3^)	3.13 ± 1.57	2.75 ± 1.65	2.35 ± 0.89
Total volume of filling of apical 3–6 mm (mm^3^)	3.10 ± 1.53	2.72 ± 1.65	2.32 ± 0.97
Total volume of voids of apical 3–6 mm (mm^3^)	0.039 ± 0.045^a^	0.032 ± 0.019^a^	0.026 ± 0.024^b^
Total % of vov in apical 0–3 mm	0.39 ± 0.30	0.48 ± 0.32	1.45 ± 0.61
Total % of vov in apical 3–6 mm	1.12 ± 0.73	1.36 ± 1.01	1.12 ± 0.69
Total % of vov	0.89 ± 0.55	1.07 ± 0.71	1.25 ± 0.59

Significant different means in the same row are indicated by different superscript letters (*P*<0.05)

### Apical microleakages

**Fig. 3:**
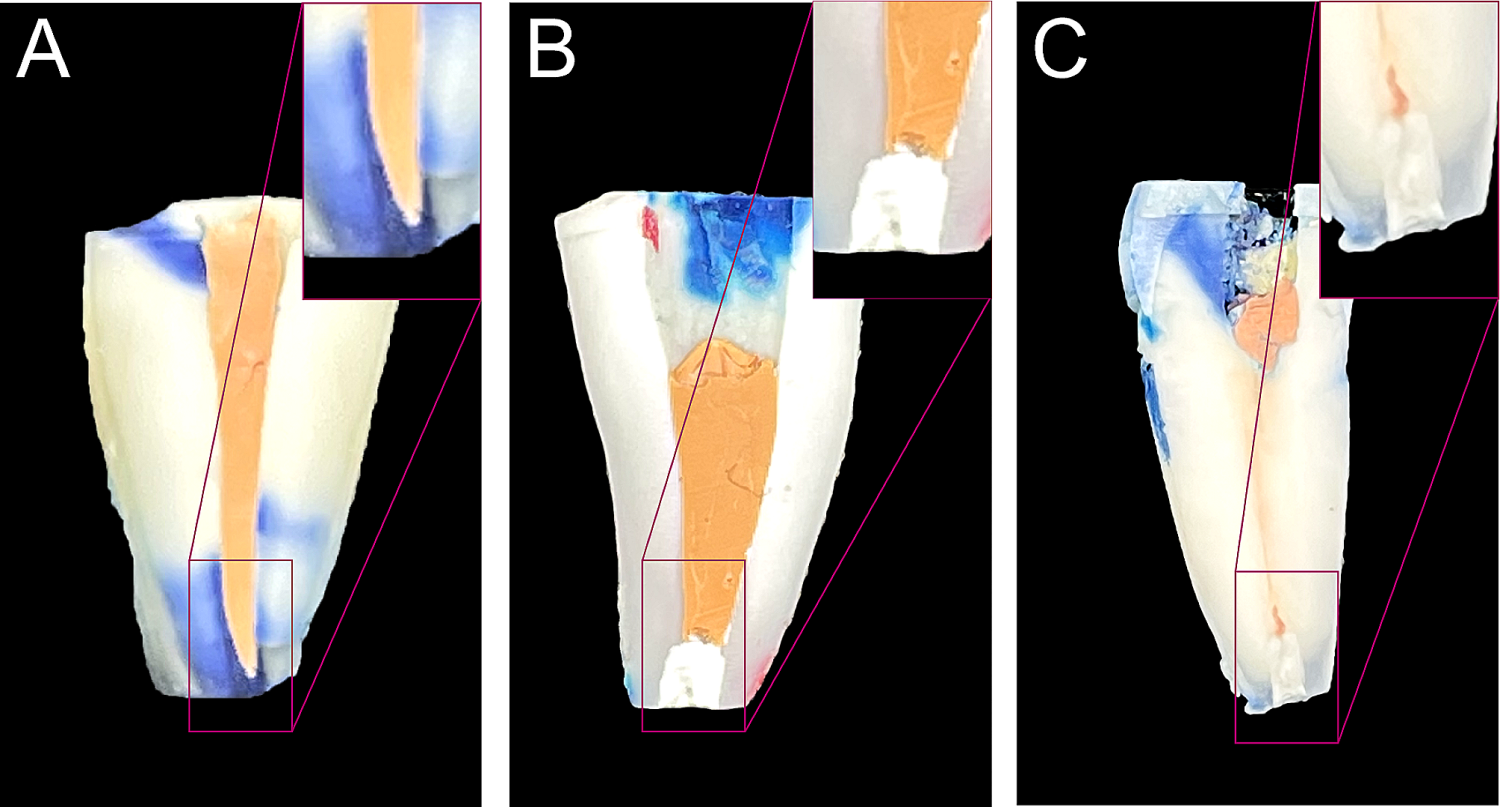
Representative pictures of microleakage assessment. **(A)** The conventional surgery group; **(B)** the standard surgery group; **(C)** the modified surgery group

**Fig. 4:**
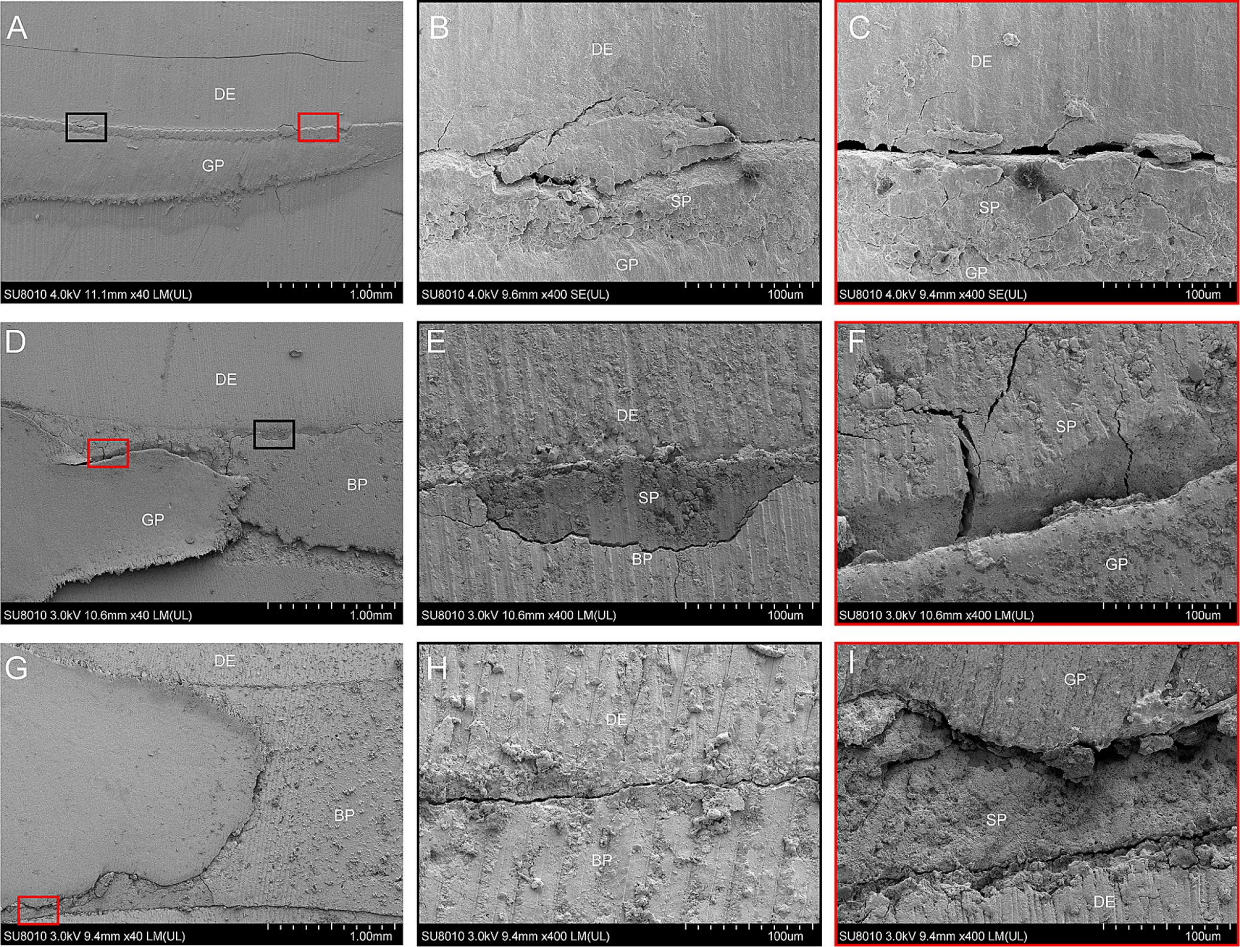
Representative SEM images of longitudinal sections. **(A)** Low-magnification images of the conventional surgery group. **(D)** Low-magnification images of the standard surgery group. **(G)** Low-magnification images of the modified surgery group. **(B), (E), (H)** High-magnification images of the black-framed areas in **(A)**, **(D)**, and **(G)**, respectively. **(C), (F), (I)** High-magnification images of black-framed areas in **(A)**, **(D)**, and **(G)**, respectively. DE, dentin; GP, gutta-percha; SP, iRoot SP; BP, iRoot BP.

**Fig. 5:**
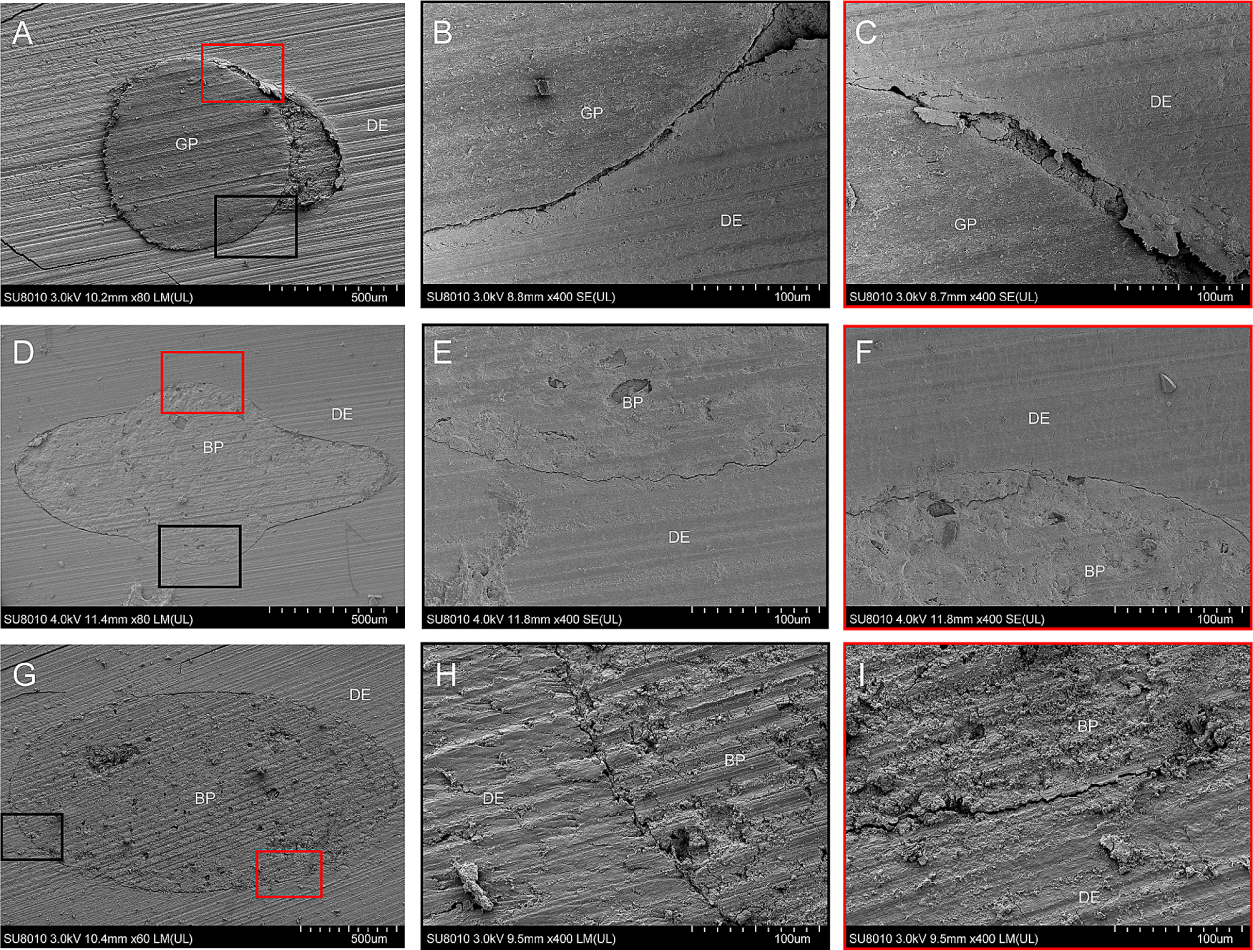
Representative SEM images of cross sections. **(A)** Low-magnification images of the conventional surgery group. **(D)** Low-magnification images of the standard surgery group. **(G)** Low-magnification images of the modified surgery group. **(K), (N), (Q)** High-magnification images of the black-framed areas in **(J)**, **(M)**, and **(P)**, respectively. **(L), (O), (R)** High-magnification images of black-framed areas in **(J)**, **(M)**, and **(P)**, respectively. DE, dentin; GP, gutta-percha; BP, iRoot BP.

Representative images of microleakages in the apical zone are presented in Fig. 3. Compared to the conventional surgery group, there was generally less microleakage in the modified surgery group and the standard surgery group. In addition, the SEM examination of longitudinal sections revealed that the fillings of the modified surgery group and the standard surgery group were closely bonded to the root canal wall, demonstrating that iRoot-BP could form reliable chemical and physical bonding with the root canal wall, whether it was orthograded or retrograded (Fig. 4D-I). However, in the conventional group, there were obvious gaps between the gutta-percha and the root canal wall (Fig. 4A-C). SEM observation of the cross sections corresponds with the results of longitudinal sections (Fig. 5). These results indicated that orthograde obturated with iRoot-BP followed by apicoectomy showed no adverse impact on the binding of iRoot-BP with the root canal wall under the premise of sufficient cure time of the filling materials.

### Representative case report

In this study, four cases of chronic periapical periodontitis in the anterior region were selected and received the modified approach. Favorable outcomes were reported in all cases. The information on the four cases is summarized in Table 2.

**Table 2:** Information of cases that received the modified approach

Sex	Age	Position of teeth*	Initial diagnosis impression	Treatment	Postoperative pathology	Follow-up time	Prognosis
Female	23	12,11,21,22	Apical periodontitis	#12,11 received the standard surgery procedure, and #21,22 received the modified surgery procedure	Lesion of #12,21 was periapical granuloma, while lesion of #21,22 was a radicular cyst	30 months	Healing
Female	28	11,12	Apical periodontitis	#12 received the standard surgery procedure, and #11 received the modified surgery procedure	Radicular cyst	29 months	Healing
Female	25	12	Apical periodontitis	The modified surgery procedure	Periapical granuloma	31 months	Healing
Female	35	11,12	Apical periodontitis	#12 received the standard surgery procedure, and #11 received the modified surgery procedure	Radicular cyst	30 months	Healing

* The positions of teeth were depicted using FDI World Dental Federation notation (ISO 3950)

**Fig. 6:**
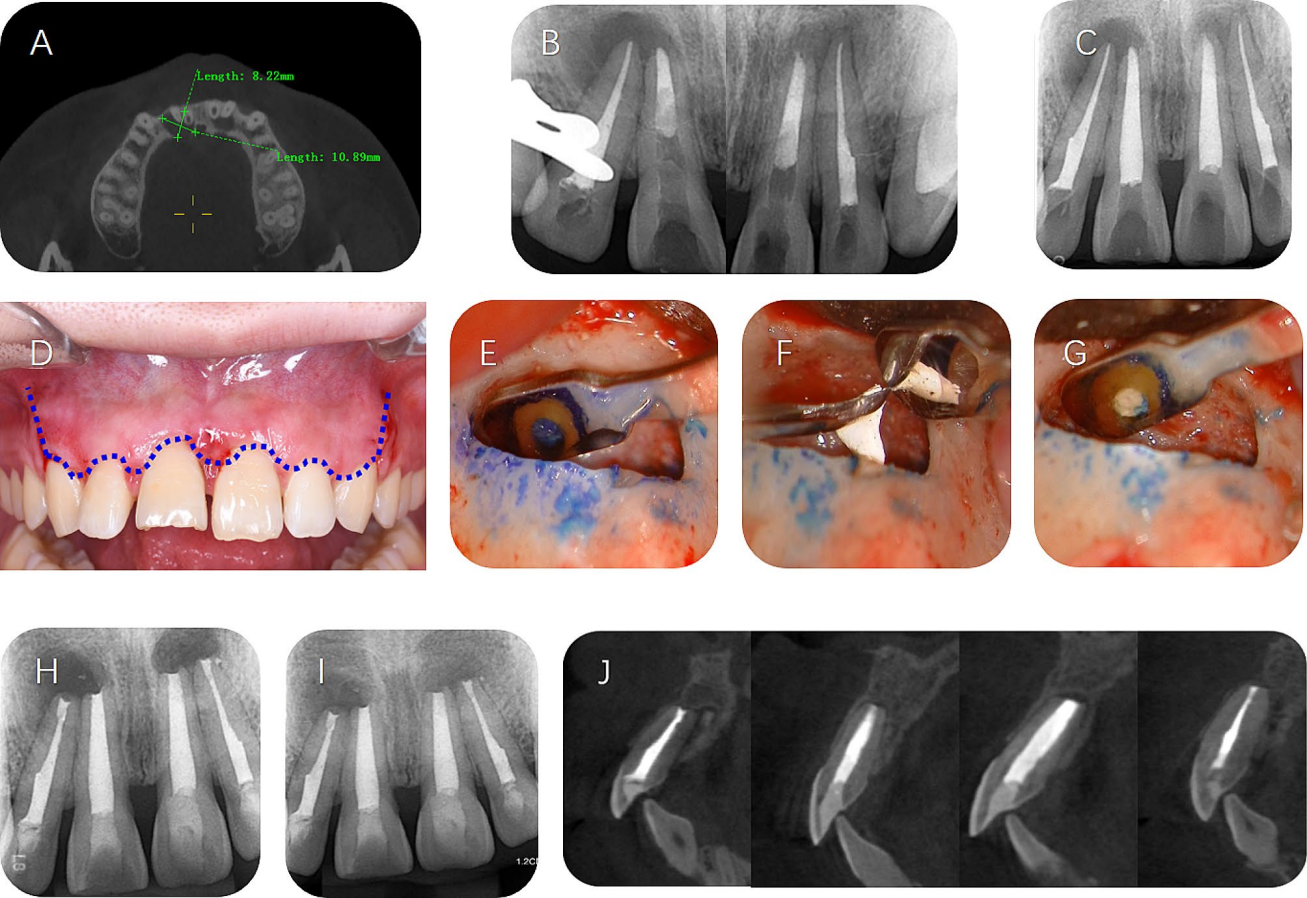
A representative case received the modified approach. **(A)** The CBCT scan revealed a lesion at the periapical region of the right central incisor and lateral incisor, and another independent lesion at the left central incisor and lateral incisor. **(B)** A periapical radiograph taken during root canal treatment indicated that the immature maxillary central incisors received orthograde obturated with iRoot-BP for 6 mm and filled with gutta-percha for the crown part, while two lateral incisors received obturation with gutta-percha and iRoot-SP by the warm gutta-percha obturation technique. **(C)** A periapical radiograph was taken at the 3-month follow-up. **(D)** A photograph taken before the surgery. The blue line represented the operative incision. **(E)**, **(F)** and **(G)** The lateral incisors received retro-preparation and retro-filling with iRoot BP after apicoectomy. **(H)** A postoperative periapical radiograph was taken immediately. **(I)** Follow-up imaging showed partially healed of lesions 3 months after surgery. **(J)** A CBCT scan at the 12-month follow-up revealed healthy apical bone tissue around all roots

One representative case of a 23-year-old woman is shown in Fig. 6, and the detailed information on diagnosis and treatment was as follows:

A 23-year-old woman without reported systemic disease or drug allergies was referred to the Department of Endodontics, the Stomatology Hospital, Zhejiang University School of Medicine, with a complaint of swelling and pain in her right upper anterior teeth for 1 year. The patient admitted a history of dental trauma 10 years ago. Clinical examination revealed that the maxillary right central incisor and lateral incisor were moderate discoloration and negative to pulp vitality test. The crown of the maxillary right central incisor was broken. The pulp of the maxillary left central incisor and lateral incisor were alive according to the pulp vitality test. Each maxillary incisor demonstrated no tenderness to percussion or palpation, respectively. The gingiva between the two central incisors showed mild swelling, and the periodontal probing was 3 mm.

The CBCT scan revealed an 11*8*7 mm oval lesion at the periapical region of the right central incisor and lateral incisor and another lesion sized 8.5*5*6 mm at the periapical region of the left central incisor and lateral incisor (Fig. 6A). Besides, both maxillary central incisors were immature with open apical foramen (diameter>2 mm) and short length (<20 mm). Therefore, a treatment option was offered, where the immature maxillary central incisors received endodontic treatment and orthograde obturated with iRoot-BP for 6 mm, while two lateral incisors received obturation with gutta-percha and iRoot-SP using the warm gutta-percha obturation technique. The patient was informed of all treatment alternatives and signed the informed consent.

Procedure of root canal treatment: A rubber dam was used to isolate maxillary incisors. Access cavities were prepared in the lingual face of each incisor to expose the pulp chamber. Root canal preparation was performed using WaveOne Gold Reciprocating Files (Dentsply Sirona), followed by 3% NaOCl solution irrigating with passive ultrasonic irrigation. Corresponding filling materials were introduced into the root canals using a hand instrument and compacted with a vertical condensation plugger. For the central incisors filled with iRoot-BP, a moist cotton pellet was placed upon the material for setting of iRoot-BP and provisionally sealed the access. An immediate postoperative X-ray radiograph was taken after iRoot-BP obturation (Fig. 6B). After a week, these central incisors were filled with gutta-percha for the crown part. Access cavities were sealed using composite resin. The patient was advised to receive follow-up examinations 3 months after completion of root canal treatment.

The periapical lesions showed no sign of healing at the 3-month follow-up, and the patient reported dull pain in the left lateral incisor during the biting and percussion examination (Fig. 6C). Combined with the consideration that there might exist cystic lesions of nonendodontic origin or neoplastic lesions together with the patients’ willingness, the surgical plan was made so that the immature maxillary central incisors received the modified surgical procedure, while the lateral incisors received the standard surgical procedure.

Local anesthesia was administered with 4% articaine hydrochloride with 1:100,000 epinephrine. Vertical incisions were made at the distal sites of the canines, and a mucoperiosteal flap was raised with intra-sulcus incisions (Fig. 6D). The periapical lesions were exposed by removing the buccal bone. Subsequently, the inflammatory granulomatous tissues were removed with a curette. The apical 3 mm of the root of each maxillary incisor was resected using a high-speed bur. The lateral incisors received retro-preparation using ultrasonic tips and retrograde filling with iRoot-BP (Fig. 6E-G), while the central incisors only received resection of 3 mm of roots. The cut surfaces of the roots were stained with methylene blue, and the quality of root canal filling was checked. No visible leakage was found. The mucoperiosteal flap was repositioned and sutured. The immediate postoperative X-ray radiograph showed compact root canal filling (Fig. 6H). The pathological diagnosis of the lesion at the periapical region of the left central incisor and lateral incisor was a radicular cyst, while the pathological diagnosis at the periapical region of the right central incisor and lateral incisor was periapical granuloma.

The follow-up time was set every 3 months after the surgery. All teeth were asymptomatic, with a negative reaction to percussion, not loosening, and healthy periodontal tissue. 3-month recalls showed signs of healing of the periapical tissues (Fig. 6I). The CBCT scan at the 12-month recall revealed healthy apical bone tissue around all roots (Fig. 6J). Moreover, radiographs and clinical images of other cases were shown in Figure S1–S3. Histological pictures of lesion tissues can be found in S4–S7. Therefore, we suggest that this simplified surgical procedure can achieve a good prognosis in vivo.

## Discussion

Currently, endodontic microsurgery is the main treatment way for refractory apical periodontitis. The standard surgical procedures require the application of iRoot BP or mineral trioxide aggregate as the retrograde filling material [3]. Microsurgery relies on expensive and specialized equipment and is highly technical sensitive. Therefore, this study proposed a novel simplified approach for endodontic microsurgery in single-rooted teeth with short length (< 20 mm) and compared the filling effects and apical microleakages of teeth that received the new approach with those received the conventional method or the current standard method.

An impeccable coronal and apical seal is of utmost importance for preventing bacterium and the by-products with three-dimensional obturation [19]. Additionally, in surgical endodontic treatments, a hermetic seal between the retrograde filling material and the root canal wall is an important factor that affects treatment outcomes [20, 21]. A systematic review elaborated on the negative and positive impacts of both types of voids on root-end filling quality [22]. However, others hold the opinion that the voids in the gap volume may have a negative effect on the filling quality, while the internal voids may not [23]. The gap between the filling materials and dentinal walls may offer the collateral channel for microorganisms. It has been found that Enterococcus faecalis [24], one of the commonly-seen bacteria in the reinfection, is approximately 2.0 μm-diameter [25], much smaller than the voids ranging from 0.004 to 0.018 mm^3^, making it possible for Enterococcus faecalis to migrate through the voids in the gap volume [26]. A previous study found that the orthograde technique and apical resection without retro-filling can achieve a similar number of voids compared to methods containing retrograde obturation in single-rooted permanent teeth [27]. Micro-CT and µCT techniques enable analysis of voids in filling materials without damaging the samples, which have been applied in a lot of studies [28, 29]. The main limitation of µCT is that the filling materials introduce artifacts in the images, which may influences quantitative analyses [30]. In this study, µCT imaging revealed that the volumes and ratios of voids in apical 0–3 mm and 3–6 mm in the modified surgery group and standard surgery group were comparable. Therefore, it is suggested that this modified surgery can achieve similar filling effects as the current standard method.

Since the voids in the gap volume may induce the reinfection in periapical tissues through the migration of the bacterium, an effective apical seal can be the last line of defense to obturate the apical hole, preventing microorganisms from encroaching the apex and apical percolation. Although the bioceramic materials are superior to the traditional canal sealers for their expansiveness, causing fewer and smaller gaps, numerous studies have shown that almost all kinds of filling materials inevitably remain gaps in the apex, resulting in microleakages [31–33]. Studies have indicated that approximately 60% of surgical failures were attributed to apical microleakages [34].

Dye penetration and SEM were applied in this study to analyze microleakages. Dye penetration was the most common approach for measuring apical or coronal microleakages of different obturation systems because it is easy to perform [35, 36]. A previous study reported some drawbacks of dye penetration method and that its results may be influenced by other factors, such as the physical process of diffusion, filtration, and capillarity [37]. SEM examination is another extensively used method [38, 39]. It produces high-resolution images of specimens and can show the adaption of materials to the dentinal walls. But both dye penetration and SEM analyses require sectioning of the root, testing methods without sample destruction are needed in future. In this study, the results revealed that the gaps in the conventional group are much more obvious than in the standard group or the modified group, as gutta-percha cannot tightly adhere to root canal walls, while bioceramic materials can form physical and chemical bonds with dentin, resulting in tight junction.

Advances in root-end filling material are essential for improving the outcomes of apical surgery/microsurgery [5]. The simplified surgical procedure in this study is based on the bioactivity, biocompatibility of calcium silicate-based bioceramics [40]. There are also other studies concerned with reducing technical sensitivity of apical microsurgery based on bioceramic materials. Recently, Dong et al. reported that combined application of iRoot-BP and iRoot-SP as root-end filling materials achieved better apical sealing effects in vitro [41]. Yang et al. found that when the orthograde filling of root canals was poor, backfilling with 3-mm iRoot-BP was not enough to achieve good apical sealing effects [42]. This may due to the difficulty in operation of retrograde filling during the surgery. The modified procedure suggested by this study can reduce the technical sensitivity of apical microsurgery, especially the difficulties related with retropreparation and retrofilling.

In vitro experiment confirmed that the modified group had similar filling and sealing performances with the standard group. However, its effect in practical application is unclear. Therefore, we selected four cases of chronic periapical periodontitis to explore the in vivo effect of this new modified surgery. All cases had achieved a good prognosis. Adham et al. also reported two successful cases that received a similar approach in which the palatal roots of maxillary molars received selective retreatment and obturation with bioceramic materials followed by resection of root without retro-preparation and retro-filling [43]. Based on the aforementioned evidence, we suggest that the modified procedure can achieve a reliable prognosis in appropriate cases.

However, there are still limitations in this study. First, the indication for this modified surgery is very narrow or specific, and in vitro, simulated surgical procedures cannot reflect realistic conditions during surgery, such as bleeding, limited visual field, and operation time. In addition, although there are four successful clinical cases reported in our study, for now, there is still a lack of evidence from prospective clinical studies. Further clinical evidences are needed.

## Conclusions

This study proposed a simplified approach for endodontic microsurgery in single-rooted teeth (< 20 mm) and found that this procedure had similar root canal filling and apical sealing abilities with the standard surgical procedure. Furthermore, the preliminary clinical application of this modified surgical procedure achieved good results. Therefore, this novel simplified approach can be a reliable alternative for single-rooted teeth with short lengths.

### Electronic supplementary material

Below is the link to the electronic supplementary material.

Supplementary Material 1

## Data Availability

No datasets were generated or analysed during the current study.
