# Supplementary material for: A novel simplified approach for endodontic retrograde surgery in short single-rooted teeth
Source: BMC Oral Health. 2024 Jan 31;24:150. doi: 10.1186/s12903-024-03879-6 (PMC10832180; doi:10.1186/s12903-024-03879-6)
Supplement: Supplementary file 1 — Supplementary Material 1 [file 12903_2024_3879_MOESM1_ESM.docx]

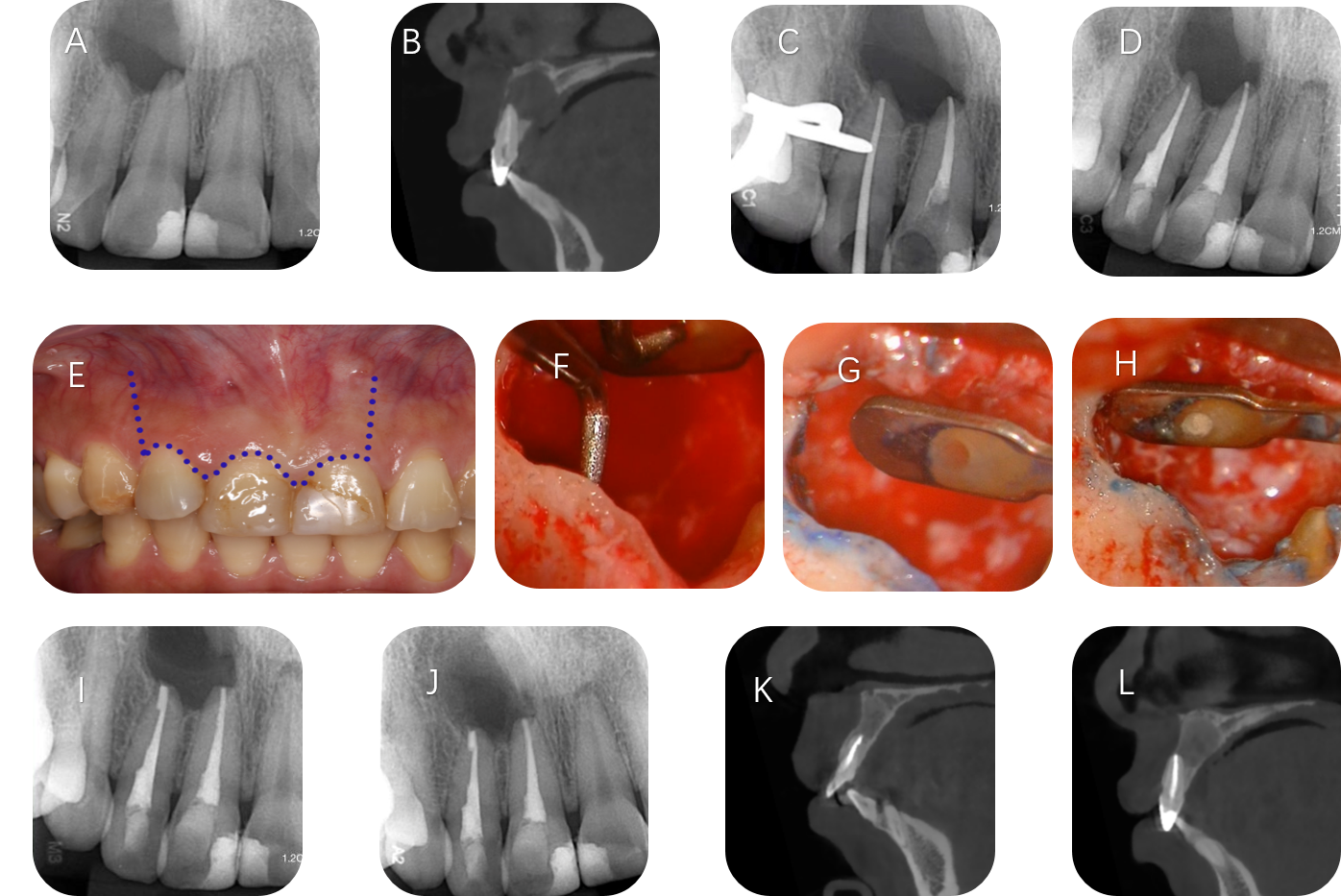


**Fig. S1** Radiographs and clinical images of case 2 (Female, 28 years old). **(A)** A preoperative radiograph. **(B)** The CBCT scan revealed a lesion at the periapical region of the right central incisor and lateral incisor. **(C)** A periapical radiograph taken during root canal treatment indicated that #12 received the standard surgery procedure, and #11 received the orthograde obturation with iRoot-BP over 6 mm. **(D)** A periapical radiograph was taken after 6 months. **(E)** A photograph taken before the surgery. The blue line represented the operative incision. **(F)**, **(G)** and **(H)** The right lateral incisor received retro-preparation and retro-filling with iRoot BP after apicoectomy. **(I)** A postoperative periapical radiograph was taken immediately. **(J)** Follow-up imaging showed partially healed of the lesion 3 months after surgery. **(K)** and **(L)** A CBCT scan at the 12-month follow-up revealed healthy apical bone tissue around all roots.


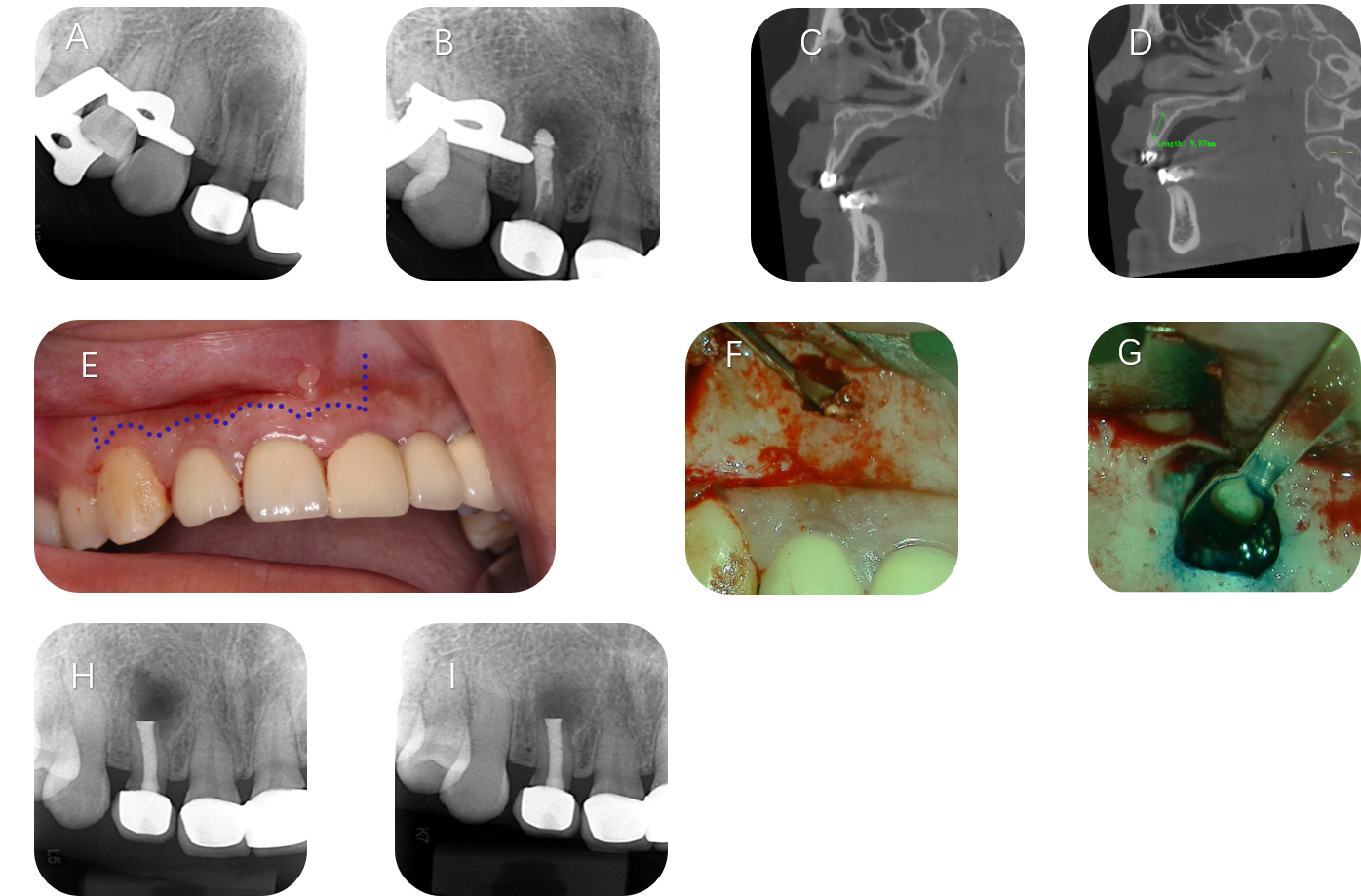


**Fig.S2** Radiographs and clinical images of case 3 (Female, 25 years old). **(A)** A periapical radiograph taken before starting root canal treatment. **(B)** A periapical radiograph taken after the right lateral incisor received orthograde obturated with iRoot-BP. **(C)** and **(D)** The CBCT scan revealed a lesion at the periapical region of the right lateral incisor. **(E)**, **(F)** and **(G)** The right lateral incisor received apicoectomy. (H) A postoperative periapical radiograph was taken immediately. **(J)** Follow-up imaging showed partially healed of lesions 3 months after surgery. The CBCT images of this case at the 12-month follow-up were absent because the patient was pregnant.


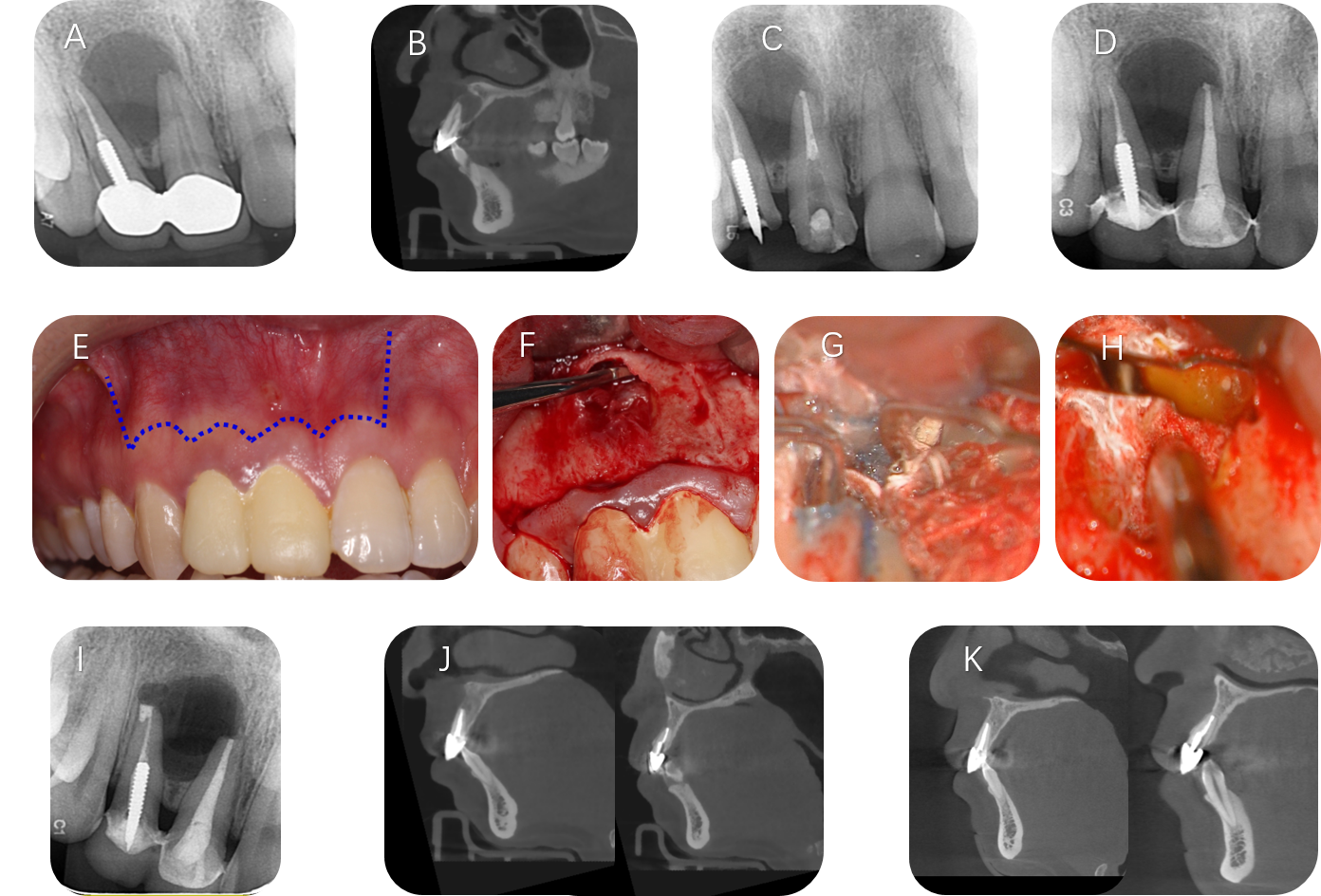


**Fig.S3** Radiographs and clinical images of case 4 (Female, 35 years old). **(A)** A preoperative radiograph. **(B)** The CBCT scan revealed a lesion at the periapical region of the right central incisor and lateral incisor. **(C)** A periapical radiograph taken during root canal treatment indicated that the right central incisor received orthograde obturation with iRoot-BP. **(D)** A periapical radiograph was taken after 6 months. **(E)** A photograph taken before the surgery. The blue line represented the operative incision. **(F)**, **(G)** and **(H)** The right lateral incisor received retro-preparation and retro-filling with iRoot BP after apicoectomy, while the central incisor only received apicoectomy. **(I)** A postoperative periapical radiograph was taken immediately. **(J)** A CBCT scan at the 12-month follow-up revealed partially healed of the lesion. **(K)** A CBCT scan at the 24-month follow-up revealed healthy apical bone tissue around all roots.


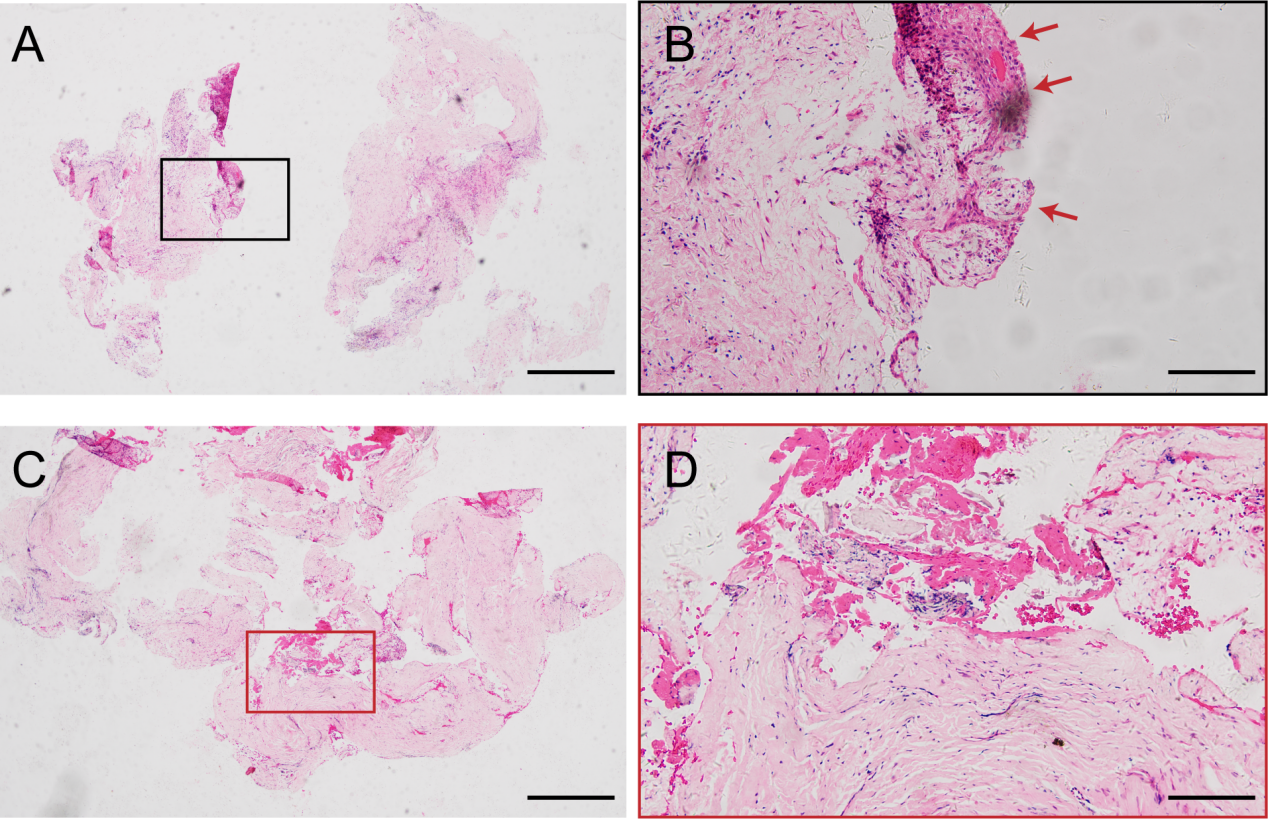


**Fig.S4** Histological pictures of apical lesion tissues from case 1 (Female, 23 years old). **(A)** The lesion of #21,22 was diagnosed as a radicular cyst (scale bar = 500 μm). **(B)** Images showing details of black-framed areas in (A). The red arrows indicating the epithelial lining (scale bar = 100 μm). **(C)** The lesion of #11,21 was diagnosed as a periapical granuloma (scale bar = 500 μm). **(D)** Images showing details of red-framed areas in (C) (scale bar = 100 μm).


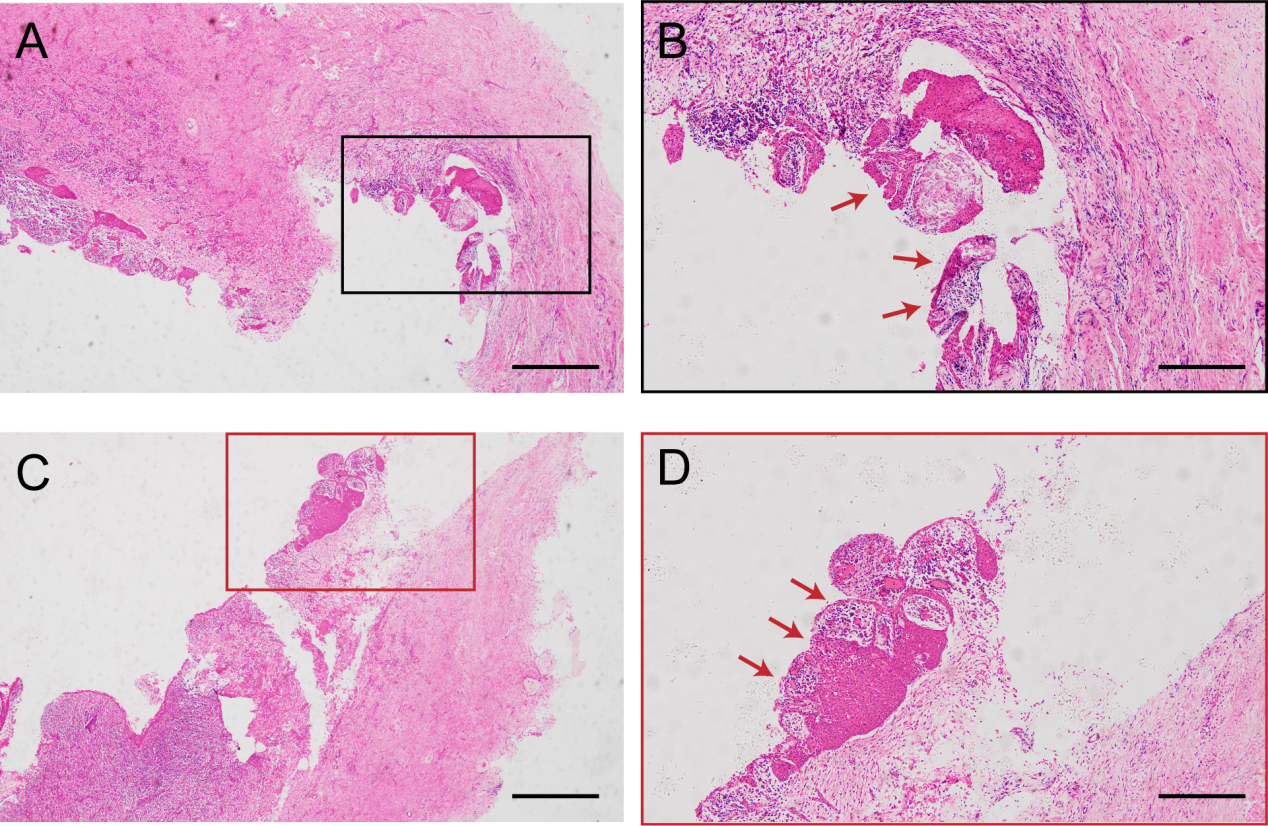


**Fig.S5** Histological pictures of the apical lesion tissue from case 2 (Female, 28 years old). **(A)** and **(C)** The lesion was diagnosed as a radicular cyst (scale bar = 500 μm). **(B)** Images showing details of black-framed areas in (A) (scale bar = 200 μm). **(D)** Images showing details of red-framed areas in (C) (scale bar = 200 μm). The red arrows indicating the epithelial lining.


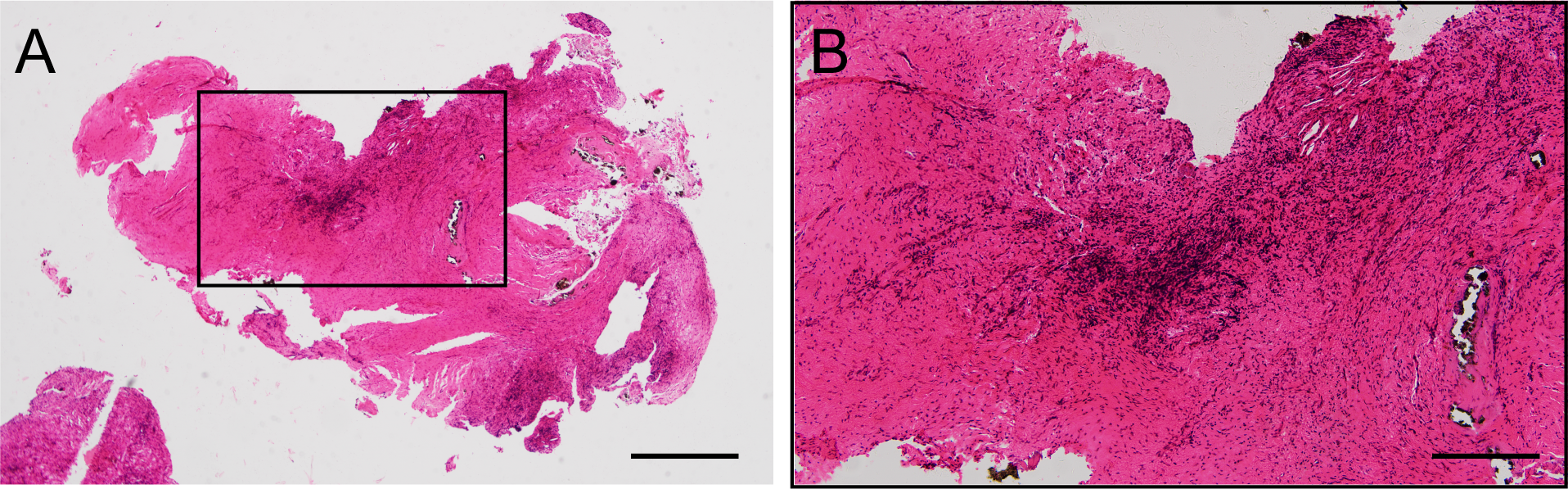


**Fig.S6** Histological pictures of the apical lesion tissue from case 3 (Female, 25 years old). **(A)** The lesion was diagnosed as a periapical granuloma (scale bar = 500 μm). **(B)** Images showing details of black-framed areas in (A) (scale bar = 200 μm).


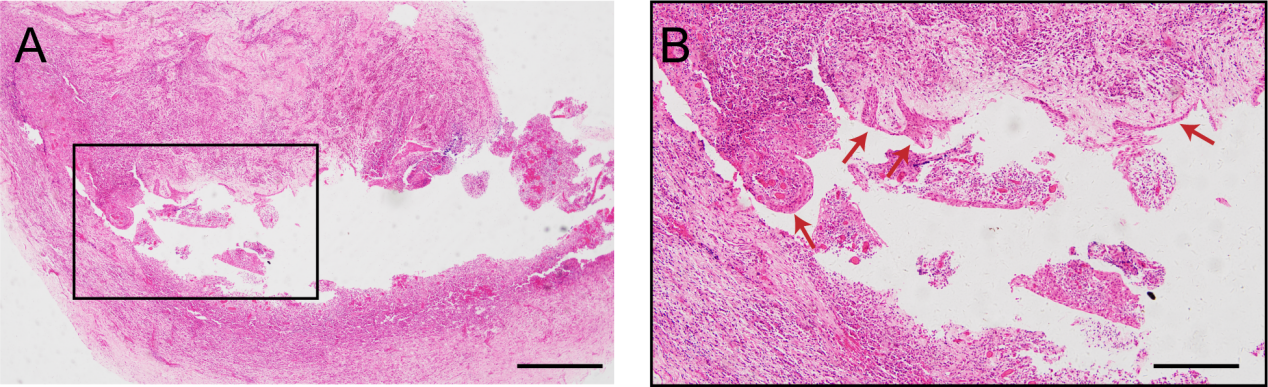


**Fig.S7** Histological pictures of the apical lesion tissue from case 4 (Female, 35 years old). **(A)** The lesion was diagnosed as a periapical cyst (scale bar = 500 μm). **(B)** Images showing details of black-framed areas in (A) (scale bar = 200 μm).The red arrows indicating the epithelial lining.
